# Editorial: Advances in breeding for waterlogging tolerance in crops

**DOI:** 10.3389/fpls.2023.1284730

**Published:** 2023-09-18

**Authors:** Ayyagari Ramlal, S. K. Lal, Vidyasagar Sathuvalli

**Affiliations:** ^1^School of Biological Sciences, Universiti Sains Malaysia (USM), Georgetown, Malaysia; ^2^Division of Genetics, Indian Council of Agricultural Research (ICAR)-Indian Agricultural Research Institute (IARI), New Delhi, India; ^3^Department of Crop and Soil Science, Oregon State University, Hermiston, OR, United States

**Keywords:** waterlogging, breeding, crops, environmental stress, tolerance

Plants continuously face and are exposed to environmental stimuli and stressors such as light, temperature, water and nutrients. Plants are sessile and must develop strategies to overcome harsh climatic conditions and stresses. Stress restricts any organism from achieving its full growth and ability. Abiotic stresses including waterlogging, heat, drought, cold and salinity are some of the primary causes of global crop losses. Waterlogging leads to anoxia; soil saturation and hypoxia ultimately resulting in a reduction in production and yield (Ahmed et al., 2013; Fukao et al., 2019; Pan et al., 2021; Mareri et al., 2022; Parrotta et al., 2023). The majority of field crops are vulnerable to water stress at some point during their growth and development. Recent estimates indicate that about 12% of the planet’s arable land is frequently affected by waterlogging, leading to yield reductions of 20%. In the near future, this could increase as a result of climate change (Tian et al., 2021). Subsequent crop failures would become crucial factors threatening the food security of an increasing global population. For instance, soybean is highly prone to waterlogging during the germination, emergence and grain-filling stages (Ploschuk et al., 2022; Rajendran et al., 2022; Rajendran et al., 2023). Staple crops such as wheat, rice, barley, maize and others are highly susceptible to flooding (Tian et al., 2019; Panda and Barik, 2021; De Castro et al., 2022; Pais et al., 2022; Zhang et al., 2023). Necrosis, stunting, defoliation, poor yield, reduced nutrient availability, and plant death are common expressions of waterlogging (Hasanuzzaman et al., 2017). Identification of crop lines of staple crops susceptible or resistant to waterlogging is crucial for the development of tolerant lines through advanced breeding approaches, with marker-assisted selection and genomic selection for the development of resistant varieties (Devi et al., 2017). Extreme environmental fluctuations challenge desirable plant performance and yield responses. Plant interactions with environmental cues shape their growth and fate. There is an urgent need to develop strategies, methods and tools to identify broad-spectrum tolerance in plants that will support sustainable crop production under hostile environmental conditions. The mechanisms by which plants perceive environmental cues and relay those signals through their molecular networks to regulate their genetic machinery for growth and survival must be elucidated and deciphered.

A review of high-impact articles providing evidence and insights on plant responses to waterlogging stress will contribute to effective approaches to the development of climate-smart food crops and waterlogging-tolerant varieties to meet the food and feed requirements of future generations.

*Taxodium ascendens* Brongn. (synonym of *Taxodium distichum* var *imbricatum* (Nutt.) Croom. [Cupressaceae] is a tree species with high tolerance to flooding, that generates knee roots in wetlands. Qian et al. investigated the number and size of knee roots and subsurface roots, and their anatomical structures, physiology, and biochemical responses at various developmental stages under conditions of soil flooding. They delineated the adaptation mechanisms of *T. ascendens* to waterlogging stress and the formation of the knee roots. Their study revealed the mechanisms of knee root formation and provided scientific evidence for afforestation and *T. ascendens* management under waterlogged conditions.

Kitao et al. explored the successful natural regeneration of *Betula platyphylla* var. *japonica* (synonym of *B. platyphylla* subsp. *mandshurica* (Regel) Kitag.; Japanese white birch) [Betulaceae] and how soil water content modifies its competitiveness against perennial weeds. They took an ecophysiological approach with greenhouse and field experiments and a field survey to investigate the competitiveness of *Eupatorium* L. [Asteraceae] species. The authors concluded not always humid soils might be favourable to the rate of photosynthesis and permit Japanese white birch to compete favourably against *Eupatorium* species.

Waterlogging during the early stages of cotton (*Gossypium* L.) [Malvaceae] growth and development has adverse effects. The improvement of cotton for better yields and quality depends on the establishment of functional relationships between growth parameters and waterlogging duration. Beegum et al. have observed that the physiological and morphological parameters of cotton (height, stem diameter, number of main stem leaves, leaf area etc.) were inversely correlated with the number of days of waterlogging stress. Biochemical factors such as a decrease in macro- and micronutrient availability showed mixed trends as days of waterlogging stress increased. Therefore, this study can serve as a basis for developing cotton models to simulate the impact of waterlogging on cotton.

The development and adoption of anaerobic germination (AG) (or AG percentage; AGP) tolerant rice varieties *Oryza sativa* L.) [Poaceae] was described by Shanmugam et al. These AG varieties are important during this era of climate change. They explored the wider genetic variation for AG potential associated traits in a panel of 115 rice germplasms and identified Karuthakar (100), Poovan Samba (96.67), Mattaikar (96.67), Edakkal (96.67), Manvilayan (93.33), Mandamaranellu (93.33) and Varappu Kudainchan (93.33) as highly tolerant landraces. Their study also identified AG-tolerant landraces whose seeds are long and strong, with high shoot and root length as well as superior AG percentage and anaerobic vigour index when compared with other grain types.

Plants experience multiple stresses simultaneously, or multifactorial stress combinations (Zandalinas and Mittler, 2022). The work on the development of smart rice for drought-waterlogging stresses by Rahman and Zhang provides useful insights into the significance of multiple stresses on the growth, development, yield and production of rice and can be extrapolated to other crops as well.

From the contributions made to this topic, The key takeaways from a brief review of this topic elucidate waterlogging tolerance mechanisms including genetic, biochemical and physiological aspects of staple crops (rice), cash crops (cotton) and trees (pioneer tree and pod cypress). We hope that this useful knowledge will facilitate new and advanced studies and breeding strategies for crops of agricultural importance experiencing waterlogging. Combined efforts among theoretical research, varietal development and field-related studies are essential if we are to understand waterlogging tolerance in crops. We are grateful for the efforts of the journal editors, peer reviewers, and authors on this topic. This volume would not be possible without their significant contributions. We hope that our readers can identify valuable information from this volume and identify appropriate collaborators to advance work in this important area.

## Author contributions

AR: Data curation, Resources, Writing – original draft, Writing – review & editing. SL: Conceptualization, Supervision, Visualization, Writing – review & editing. VS: Conceptualization, Supervision, Visualization, Writing – review & editing.
